# Effects of Foliar Spraying of Dicarboxylicdimethylammonium Chloride on Cadmium and Arsenic Accumulation in Rice Grains

**DOI:** 10.3390/toxics12060418

**Published:** 2024-06-07

**Authors:** Lin Fu, Jiawei Deng, Dayliana Ruiz Lao, Changbo Zhang, Weijie Xue, Yun Deng, Xin Luo

**Affiliations:** 1 Key Laboratory of Original Agro-Environmental Pollution Prevention and Control, Ministry of Agriculture and Rural Affairs, Agro-Environmental Protection Institute, Ministry of Agriculture and Rural Affairs, Tianjin 300191, China; fl1004197187@163.com (L.F.); d17736218037@163.com (J.D.); 18501338578@163.com (D.R.L.); l_xin01@163.com (X.L.); 2 School of Environment and Ecology, Jiangnan University, Wuxi 214122, China; dengyun@jiangnan.edu.cn

**Keywords:** foliar inhibitor, dicarboxylicdimethylammonium chloride, rice, cadmium, arsenic, accumulation

## Abstract

A field experiment with double cropping rice was carried out to study the foliar application effects of dicarboxylicdimethylammonium chloride (DDAC) on cadmium (Cd) and arsenic (As) accumulation in rice grains. The results showed that the spraying of DDAC could significantly reduce the accumulation of Cd and As in rice grains. The highest reductions in Cd and As content were observed when 1.5 mmol L^−1^ DDAC was sprayed, with 49.1% and 27.4% reductions in Cd and As content in early rice grains and 56.5% and 28.1% reductions in Cd and As content in late rice grains, respectively. In addition, the content of calcium (Ca) in rice grains increased significantly after DDAC foliar application, which was also conducive to the synthesis of amino acids such as glutamate (Glu), glycine (Gly) and cysteine (Cys) in rice grains. The results indicated that the foliar spraying of DDAC can inhibit the absorption, transport, accumulation and toxicity of Cd and As in rice grains by increasing amino acid synthesis and regulating the absorption and transport of essential elements.

## 1. Introduction

As two non-essential-harmful elements, cadmium (Cd) and arsenic (As) are easily absorbed by rice through the soil-plant system and are transported to grains for accumulation [1,2]. Emissions from industry and mining, as well as the misuse of fertilizers and pesticides, lead to widespread Cd and As production and their co-contamination of paddy soil in China, posing a threat to food security and human health [3,4,5]. 

Due to the opposite environmental behaviors of Cd and As in soil, the synchronous control of Cd-As composite pollution is extremely challenging [6]. Water management is widely used in the remediation of Cd-As composite pollution, but the effectiveness of joint governance is often unable to be achieved due to the inability to strictly control soil pH and Eh [7,8]. Treatment methods involving the addition of Cd and As passivators to soil often pose the risks of reactivation and secondary pollution [9], and 50–70% of the Cd and As in rice grains mainly comes from the remigration and reactivation of Cd and As accumulated in vegetative organs before the flowering period [10,11]. Therefore, inhibiting the activity of Cd and As in vegetative organs is one of the most effective pathways for the simultaneous remediation of co-contamination with Cd and As, such as in foliar spraying technology, which can inhibit the remigration and reactivation of heavy metals [12,13]. 

The transport of Cd in plants is usually carried out by metal ion transporters or cation transport channels such as K^+^, Ca^2+^, Mg^2+^, Mn^2+^, Fe^2+^, and Zn^2+^, which leads to a competitive relationship between Cd and these cations [14]. The exogenous addition of K, Ca, Mn, Fe, or Zn significantly increases the absorption and transport of essential cations by rice, while reducing the absorption and transport of Cd [15]. The absorption pathway of As in rice is related to its current form, which can be divided into silicate (Si) transporters and phosphate (P) transporters, leading to competition between As and Si and P [16]. Essential elements are necessary for plant metabolism because they act as enzyme regulators, cofactors, and activators [17]. Furthermore, the transport rate of beneficial elements in plants is much higher than that of harmful elements [6]. Therefore, increasing the absorption of essential elements that have antagonistic effects on Cd and As may be an important method of inhibiting the absorption of Cd and As by rice.

Amino acids are the basic units of proteins, as well as the precursors of small molecules such as antioxidants and signaling metabolites, which are closely related to plant growth metabolism, the abiotic stress response, and element absorption and transport [18,19,20]. As the central substance of amino acid metabolism, glutamate (Glu) plays an important role in alleviating the toxicity of Cd and As. Studies have shown that the exogenous application of Glu downregulates the expression levels of Cd transporter-related genes in roots and alleviates Cd-induced chlorosis and growth inhibition, which demonstrates that any pathway that increases endogenous Glu content has the potential to reduce heavy metal uptake in rice and to alleviate heavy metal toxicity [21]. The exogenous application of Glu to rice under As stress protects photosynthetic function and the growth of rice plants by affecting nitrogen assimilation, proline metabolism, and the antioxidant system [22]. Unfortunately, the water solubility of Glu is extremely low, so direct foliar spraying of Glu has no significant effect on the endogenous Glu content in rice.

**Scheme 1:**
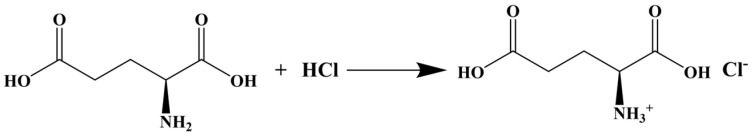
Synthetic form of DDAC.

In order to increase the water solubility of Glu, an ionic liquid named dicarboxylicdimethylammonium chloride (DDAC) was synthesized using Glu as the raw material, which has the characteristics of good environmental protection, strong water solubility, and high stability (Scheme 1). Ionic liquids with glycine as a precursor have been shown to effectively reduce Cd accumulation in rice seedlings [23,24]. However, there are no reports on the effect of DDAC on the regulation of Cd and As accumulation in rice. Therefore, a paddy field experiment was conducted to explore the effects of the foliar application of DDAC on the content of Cd, As, essential elements, and total amino acids in rice grains under Cd-As co-contamination. The results showed that DDAC could effectively reduce the accumulation of Cd and As in rice grains by regulating the absorption and transport of essential elements and promoting the synthesis of amino acids.

## 2. Materials and Methods

### 2.1. Material Preparation

In hydrochloric acid solution, the same molar number of glutamate was added and stirred evenly. The liquid was transferred to a round-bottom flask after being kept at a constant temperature reaction at 60 °C for 2 h, and was then distilled under reduced pressure with a rotary evaporator to obtain a white solid powder at room temperature—that is, DDAC.

### 2.2. Experimental Design

The field trial was conducted in Xiangtan City, Hunan Province of China (N: 27°36′, E: 112°58′), with two seasons of early rice and late rice. The test soil was Cd-As co-contaminated soil with a Cd content of 1.29 mg kg^−1^ and an As content of 39.29 mg kg^−1^. Early rice was sown in late April, sprayed in mid-June, and harvested in mid-late July. Late rice was sown in late July, sprayed in mid to late September, and harvested in November. The experiment was designed as a randomized complete block design with four replications for each treatment. Based on the results of preliminary studies and tests, different concentrations of DDAC solutions were sprayed at the flowering stage of the rice as follows: CK (control group), T1 (0.20 mmol L^−1^), T2 (0.50 mmol L^−1^), T3 (0.80 mmol L^−1^), T4 (1.20 mmol L^−1^), and T5 (1.50 mmol L^−1^) [24]. Each spray treatment was performed once a day for two consecutive days. Field management was basically consistent with that of local high-yield rice fields. Samples were collected from rice plants at maturity and dried to determine Cd, As, essential element and amino acid levels in the grains.

### 2.3. Determination of Cd and As Content

The determination of Cd and As content was completed according to previous reports [10,25]. After accurately weighing 0.5 g of the rice grains into the digestion tube, 7.0 mL of MOS HNO_3_ was added and left for more than 5 h. DigiBlock ED54 (LabTech, Beijing, China) was used for digestion at 110 °C for 2.5 h. After cooling to room temperature, 1.0 mL of H_2_O_2_ was added to continue the digestion process for 1.5 h. Finally, the temperature was raised to 170 °C for acid removal. Deionized water was added to the digestion solution to 25.0 mL, which was then filtered for the detection of Cd content. Inductively coupled plasma mass spectrometry iCAP Q ICP-MS (Thermo Scientific, Waltham, MA, USA) was used for Cd determination. The processing method of As determination was similar to that of Cd, except that the digestion temperature was fixed at 110 °C for 4 h and H_2_O_2_ was no longer added. An atomic fluorescence spectrometer AFS-8520 (Haiguang, Beijing, China) was used to determine the As content. Standard reference material (Rice Powder Certified Reference Material GBW(E)100350) and blank digestion samples were used for quality assurance and quality controls (QA/QCs). The recovery rate was 90–105% to ensure the accuracy and trustworthiness of the data. 

### 2.4. Determination of Essential Element Content

The determination of the essential element content was completed according to previous reports [26]. ICP-MS was also used to determine the content of K, Ca, Mg, Fe, Mn and Zn, and the sample treatment method was the same as in Section 2.2.

### 2.5. Determination of Total Amino Acid Content

The extraction and identification of amino acids were completed on the basis of previous reports [27]. After accurately weighing 0.25 g grain powder in a test tube, 15.0 mL of 6 mol L^−1^ HCl was added. The test tube was placed on ice to cool for 5 min, filled with high-purity nitrogen, sealed, and placed in a 110 °C constant-temperature air drying oven for 22 h. The acid solution was cooled to room temperature and filtered, and then deionized water was added to 50.0 mL; 1.0 mL of the above solution was transferred to a clean test tube and dried with nitrogen in a 50 °C water bath. The steps were repeated with 1.0 mL of deionized water. Finally, the precipitate was dissolved in sodium citrate buffer (pH = 2.2) and filtered with a 0.22 µm filter membrane (JIN TENG, Tianjin, China) for detection. The total amino acid content was determined using an Agilent 1200 high-performance liquid chromatograph (Agilent Technologies, Palo Alto, CA, USA). An advance Bio AAA column (100 mm × 4.6 mm, 2.7 µm, Agilent Technologies, USA) and diode array detector (DAD) were used.

### 2.6. Statistical Analysis

All data were expressed as mean ± standard deviation. One-way analysis of variance (ANOVA) and Duncan’s tests were used to analyze the significant differences between treatments (*p* < 0.05). Microsoft Excel 2021 and SPSS 26.0 were used for data collation and analysis and Origin 2021 was used for data visualization.

## 3. Results

### 3.1. Effects of DDAC on the Acumulation of Cd and As in Rice Grains

**Figure 1:**
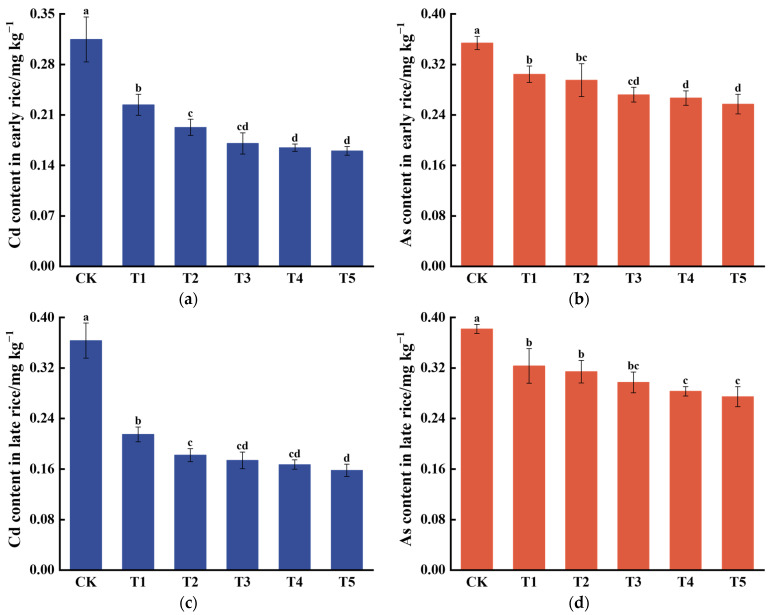
Effects of different treatments on Cd and As content in the grains of early rice (**a**,**b**) and late rice (**c**,**d**). CK, T1, T2, T3, T4, and T5 represent spray treatments of 0, 0.2, 0.5, 0.8, 1.2, and 1.5 mmol L^−1^ DDAC, respectively. Error bars represent SD. Different letters (a–d) indicate significant differences (*p* < 0.05) between treatments.

The content of Cd and As in the rice grains decreased significantly after foliar spraying of DDAC at flowering stage (Figure 1). Compared with CK, the Cd content in early rice grains decreased from 0.31 mg kg^−1^ to 0.22–0.16 mg kg^−1^, with a decrease of 28.8–49.1% when DDAC was sprayed at different concentrations. The As content decreased from 0.35 mg kg^−1^ to 0.30–0.26 mg kg^−1^ by 14.0–27.4%. The accumulation of Cd and As in late rice was higher than that in early rice. In the control group, the Cd and As content in late rice grains was 0.36 mg kg^−1^ and 0.38 mg kg^−1^, respectively. After the foliar spraying of DDAC, the Cd content decreased to 0.21–0.16 mg kg^−1^, with a decrease of 40.9–56.5%, and the As content decreased to 0.32–0.27 mg kg^−1^, with a decrease of 15.4–28.1%. In summary, DDAC, as a foliar inhibitor, can effectively alleviate both Cd and As accumulation in rice grains in Cd-As-contaminated areas. In addition, the mitigation effect of DDAC on Cd and As accumulation generally increased with increases in its concentration.

### 3.2. Effects of DDAC on the Essential Element Content of Rice Grains 

**Figure 2:**
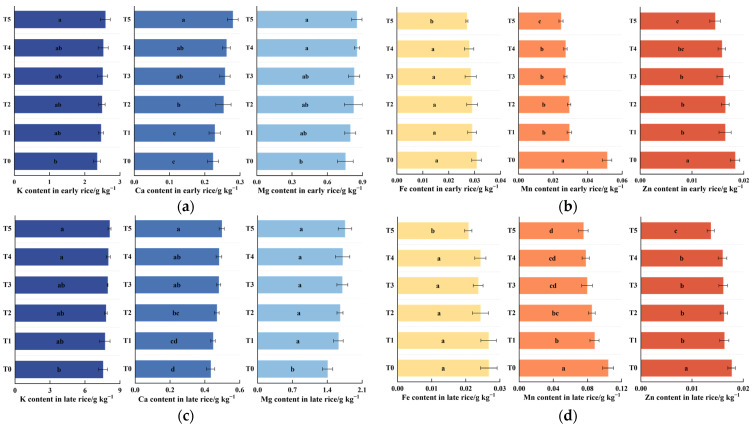
Effects of different treatments on essential element content in the grains of early rice (**a**,**b**) and late rice (**c**,**d**). T0, T1, T2, T3, T4, and T5 represent spray treatments of 0, 0.2, 0.5, 0.8, 1.2, and 1.5 mmol L^−1^ DDAC, respectively. Error bars represent SD. Different letters (a–d) indicate significant differences (*p* < 0.05) among treatments.

The application of DDAC had a significant effect on the content of essential elements in rice grains (Figure 2). Interestingly, the contents of K, Ca, Mg, and Mn in rice varied with different rice varieties and seasons, and the contents of these elements in late rice were significantly higher than those in early rice. In both early and late rice, the contents of essential elements were in the order of K > Mg > Ca > Mn > Fe > Zn. Compared with CK, the contents of K, Ca, and Mg were significantly increased after DDAC spraying, while the contents of Fe, Mn, and Zn were significantly decreased. When 1.5 mmol L^−1^ DDAC was sprayed, the contents of K, Ca, and Mg in early rice grains increased by 9.2%, 20.1%, and 11.4%, and the contents of Fe, Mn, and Zn decreased by 12.2%, 52.1%, and 21.1%, respectively. The contents of K, Ca, and Mg in late rice grains increased by 6.9%, 12.8%, and 19.8%, respectively, and the contents of Fe, Mn, and Zn decreased by 22.5%, 27.8%, and 23.0%, respectively.

**Figure 3:**
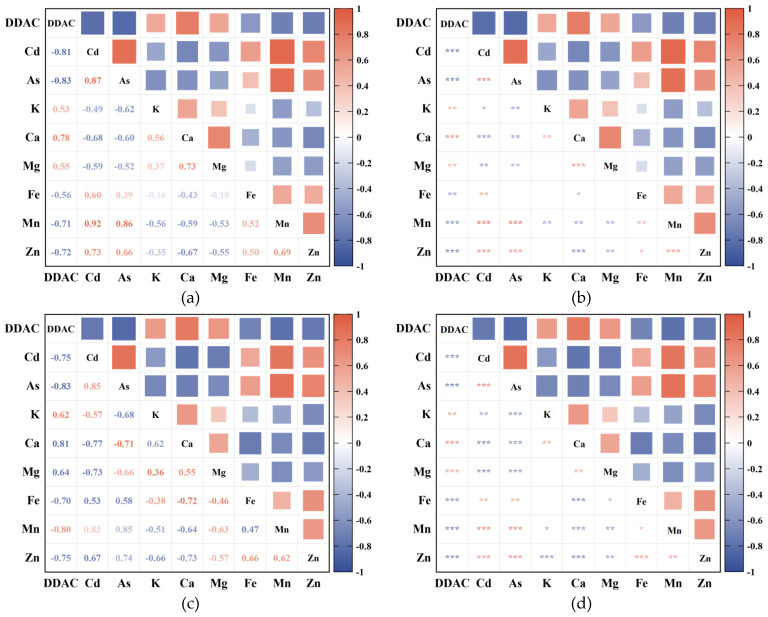
Person’s correlation analysis of Cd, As, K, Ca, Mg, Fe, Mn, and Zn content in early rice (**a**,**b**) and late rice (**c**,**d**) upon different DDAC treatments. “*” in the panel means that the correlations were significant at *p* < 0.05, “**” means that they were significant at *p* < 0.01, and “***” means that they were significant at *p* < 0.001.

In order to further explore the relationship between exogenous DDAC, Cd, As, and the essential element content, Pearson correlation analysis was performed (Figure 3). The results showed that the spraying of DDAC had significant effects on the contents of Cd, As, K, Ca, Mg, Fe, Mn, and Zn in early and late rice grains. The content of Cd in early rice grains was negatively correlated with K (r = −0.49 *), Ca (r = −0.68 ***) and Mg (r = −0.59 **), while it was positively correlated with As (r = 0.87 ***), Fe (r = 0.60 **), Mn (r = 0.92 ***), and Zn (r = 0.73 ***). The As content was not significantly correlated with Fe, but was negatively correlated with K (r = −0.62 **), Ca (r = −0.60 **), and Mg (r = −0.52 **) and positively correlated with Mn (r = 0.86 ***) and Zn (r = 0.66 ***). The correlation between elements in late rice was similar to that in early rice. The content of Cd in late rice grains was negatively correlated with K (r = −0.57 **), Ca (r = −0.77 ***), and Mg (r = −0.73 ***), while it was positively correlated with As (r = 0.85 ***), Fe (r = 0.53 **), Mn (r = 0.83 ***), and Zn (r = 0.67 ***). The As content was negatively correlated with K (r = −0.68 ***), Ca (r = −0.71 ***), and Mg (r = −0.66 ***) and positively correlated with Fe (r = 0.58 **), Mn (r = 0.85 ***), and Zn (r = 0.74 ***).

### 3.3. Effects of DDAC on the Total Amino Acid Content in Rice Grains

**Figure 4:**
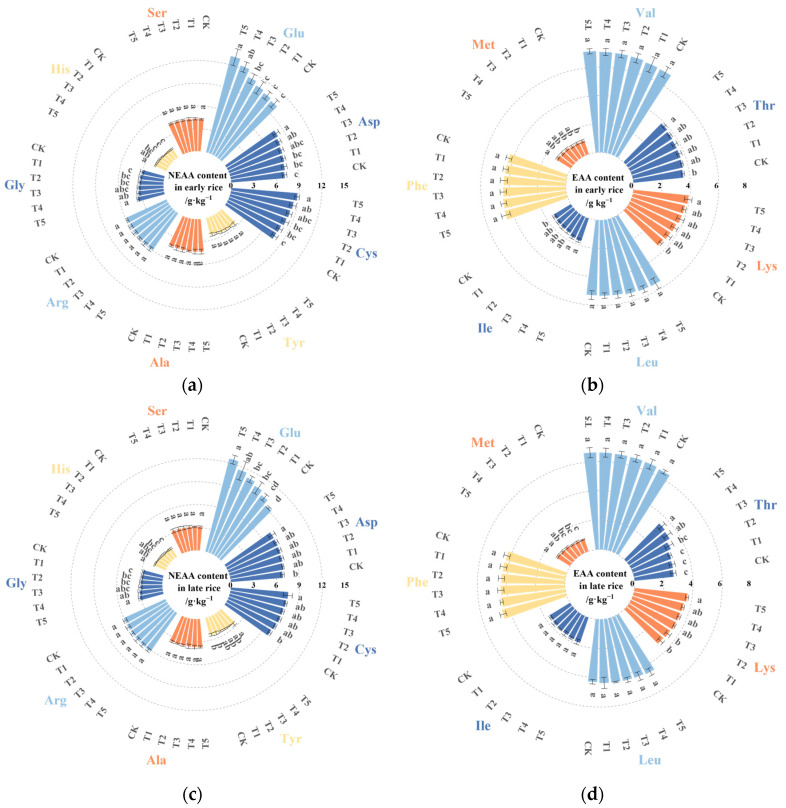
Effects of different treatments on the content of NEAA and EAA in the grains of early rice (**a**,**b**) and late rice (**c**,**d**). CK, T1, T2, T3, T4, and T5 represent spray treatments of 0, 0.2, 0.5, 0.8, 1.2, and 1.5 mmol L^−1^ DDAC, respectively. Error bars represent SD. Different letters (a–d) indicate significant differences (*p* < 0.05) between treatments. Ala, alanine; Arg, arginine; Asp, aspartate; Cys, cysteine; Gly, glycine; Glu, glutamate; His, histidine; Ile, isoleucine; Leu, leucine; Lys, lysine; Met, methionine; Phe, phenylalanine; Ser, serine; Thr, threonine; Tyr, tyrosine; Val, valine.

Spraying DDAC onto the leaf surface could increase the amino acid content in rice grains (Figure 4). Amino acids (AAs) are divided into essential amino acids (EAAs) and non-essential amino acids (NEAAs), which are important indexes for evaluating the nutritional quality of rice. The amino acid content of early rice and late rice also showed a certain difference; the total amount of NEAAs was higher than the total amount of EAAs. The amino acid content of early rice and late rice was also different; Glu was the most abundant amino acid in rice, and its content in early rice and late rice reached 10.32–13.16 g kg^−1^ and 9.29–12.57 g kg^−1^, respectively. In addition, the contents of cysteine (Cys), aspartate (Asp), and valine (Val) were also relatively rich, reaching 7.72–8.81 g kg^−1^, 7.12–7.98 g kg^−1^, and 6.89–7.14 g kg^−1^ in early rice and 6.96–7.56 g kg^−1^, 6.86–7.34 g kg^−1^, and 6.46–6.63 g kg^−1^ in late rice, respectively. Methionine (Met) and histidine (His) were the two least abundant amino acids, at only 1.01–1.18 g kg^−1^ and 1.38–1.65 g kg^−1^ in early rice and 0.87–1.06 g kg^−1^ and 1.35–1.60 g kg^−1^ in late rice. 

After DDAC spraying, the contents of Glu, Asp, Cys, glycine (Gly), and His in NEAAs in early rice were significantly increased compared to the control group, with the highest increases of 21.6%, 10.6%, 12.3%, 15.1%, and 16.3%, respectively. However, threonine (Thr), lysine (Lys), isoleucine (Ile), and Met in the EAAs increased significantly only when DDAC was sprayed at a higher concentration. Other amino acid contents did not change significantly. In late rice, Glu, Gly, His, Thr, and Met significantly increased after the spraying of DDAC, with the highest increases of 26.1%, 13.5%, 15.6%, 21.1%, and 17.3%, respectively. While the changes in Asp and Cys were significant under 1.50 mmol L^−1^ DDAC treatment. 

**Figure 5:**
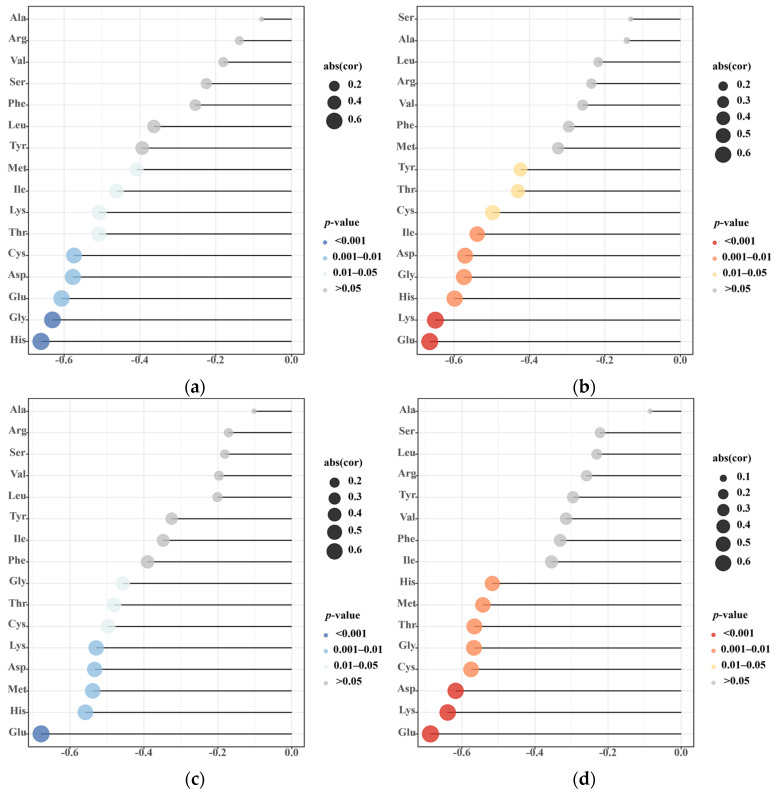
Pearson’s correlation analysis of Cd and As content and amino acid content in early rice (**a**,**b**) and late rice (**c**,**d**). There was significant correlation between the two groups when *p* < 0.05. Abbreviations have the same meaning as in Figure 4.

The correlation analysis of Cd and As content and amino acid content showed that the accumulation of Cd and As in rice was negatively correlated with the content of amino acids (Figure 5). In particular, the Glu content was strongly correlated with both the Cd and As content. The His, Gly, Glu, Asp, Cys, Thr, Lys, Ile, and Met content were significantly correlated with Cd content in early rice. The As content was no longer significantly correlated with the Met content, but was significantly correlated with Tyr. The correlation between Cd and amino acids in late rice was similar to that in early rice. The As content and Met content also showed significant correlations in late rice.

## 4. Discussion

K, Ca, Mg, Fe, Mn, and Zn are all essential nutrients required by rice, and their absorption and transport are crucial for growth, metabolism, and development in rice [28]. The transportation of these essential cations is regulated by *OsNramp*, *OsIRT*, *OsHMA*, *OsZIP*, *OsLCT*, and other transporters, as well as non-selective cation channels (NSCCs) [29,30,31]. NSCCs are a collection of channel proteins located in the inner membrane of cells such as the plasma membrane and vacuole membrane, which mediate the transmembrane transport of cations with different valence states [32]. In addition, these essential cation-related transporters and NSCCs also simultaneously regulate the absorption and transport of Cd in rice. The transport of As in rice also relies on transporters of essential elements, such as P transporters (Phts), and Si transporters (*OsLsi1* and *OsLsi2*) [33,34,35]. While the competitive effect of essential elements on transporters and channels is greater than that of harmful elements, the transport rate of beneficial elements in plants is much higher than that of harmful elements. In this study, spraying DDAC on the leaf surface significantly decreased the Cd and As content, but significantly increased the Ca content. Due to the competitive relationship between Cd and Ca, this increase in Ca content could inhibit the absorption and transport of Cd, thereby reducing the accumulation of Cd in rice grains. Studies have shown that an increase in Ca reduces the activity of As [36]. These results indicate that DDAC application can inhibit the absorption and transport of Cd by regulating the absorption and transport of essential elements, which may be related to increasing the ability of Ca to compete with transporters and NSCCs. In addition, increases in Ca can inhibit the activity of As and reduce the transport of As.

Amino acids are related to ion transport and also play a key role in the detoxification of heavy metals [37,38]. Glu and Asp are aliphatic amino acids, which can form a ring complex with Cd through two O atoms from α-COO- and the side chain-COO-. Arginine (Arg) is a basic amino acid that can form a complex with Cd through the N and O of cyanide and carboxyl groups, thereby reducing Cd activity [39]. Glu, Gly, and Cys are precursor substances for the synthesis of glutathione (GSH, γ-Glu-Cys-Gly) and phytochelatins (PCs, γ-Glu-Cys)_n_-Gly) [40,41]. GSH and PCs form chelates with Cd via S atoms on the sulfhydryl group, thereby reducing the activity of Cd and inhibiting the migration and transport of Cd [42]. Most plants have a strong ability to reduce As(V), and As(V) entering plant cells can be rapidly reduced to As(III) by arsenate reductase [43,44]. As(III) can form complexes with GSH and PCs, which are further isolated into the vacuole by the ABBC1/ABCC2 transporter [45]. In this study, the foliar spraying of DDAC promoted the synthesis of amino acids and alleviated amino acid metabolism disorder induced by Cd-As co-contamination. Increases in Glu, Gly, Cys, Asp, and Arg content were conducive to the synthesis of GSH. GSH, Glu, Asp, and Arg can be used as ligands to promote the chelation of heavy metals. These results indicate that the foliar spraying of DDAC increased the chelation of chelating ligands with Cd and As in rice, thereby jointly inhibiting the accumulation of Cd and As in rice grains.

In addition, the selective permeability of glutamate receptor-like channels (GLRs) is regulated by ligands and is closely related to Ca conduction and cation transport, and its selectivity for beneficial elements is much higher than that for harmful elements [46,47]. Glu, Gly, Cys, Asp, alanine (Ala), and serine (Ser) can be used as activators for GLRs to regulate the flow rate of ions entering and leaving channels [48,49]. Research has shown that the higher the activity of GLRs, the stronger the ability of rice to inhibit Cd transport, and rice with damaged GLR structures lead to excessive Cd accumulation in rice grains [50]. These results indicated that increasing GLR activity could significantly reduce the uptake and transport of Cd in rice. In this study, the leaf spraying DDAC was able to effectively increase the content of amino acids such as Glu, Asp, Ala, and Ser, which can be used as GLR activators, and may improve the recognition ability of GLRs for harmful elements, thus inhibiting the absorption and transport of Cd. 

## 5. Conclusions

The accumulation of Cd and As in rice grains can affect the absorption of essential elements and the synthesis of amino acids. Field experiments were conducted to investigate the effects of foliar spraying of DDAC on Cd and As accumulation in rice grains in Hunan province. The results indicated that the foliar spraying of DDAC could significantly reduce the Cd and As content in rice grains. At the same time, the content of Ca in the grains was significantly increased. In addition, Glu, Gly, Cys, and other amino acid contents were significantly increased with the foliar spraying of DDAC; this was beneficial for rice, alleviating the toxicity caused by Cd and As. In conclusion, the foliar spraying of DDAC can promote amino acid synthesis and regulate the absorption and transport of essential elements in rice. Therefore, the accumulation of Cd and As in rice grains was significantly reduced. Foliar application of DDAC provides a new idea and method for solving the problem of Cd and As exceeding acceptable standards in rice.

## Data Availability

Dataset available on request from the authors.
